# Considerations on dosimetry for in vitro assessment of e-cigarette toxicity

**DOI:** 10.1186/s12931-022-02286-1

**Published:** 2022-12-17

**Authors:** Valérie Forest, Clément Mercier, Jérémie Pourchez

**Affiliations:** Mines Saint-Etienne, Univ Jean Monnet, INSERM, U1059 Sainbiose, Centre CIS, 158 Cours Fauriel, CS 62362, 42023 Saint-Etienne Cedex 2, France

**Keywords:** In vitro inhalation toxicology, E-cigarette toxicity, Dosimetry, Physiological doses

## Abstract

Electronic cigarettes (or e-cigarettes) can be used as smoking cessation aid. Some studies tend to show that they are less hazardous than tobacco cigarettes, even if it does not mean they are completely safe. The huge variation in study designs assessing in vitro toxicity of e-cigarettes aerosol makes it difficult to make comparisons and draw robust and irrefutable conclusions. In this paper, we review this heterogeneity (in terms of e-cigarette products, biological models, and exposure conditions) with a special focus on the wide disparity in the doses used as well as in the way they are expressed. Finally, we discuss the major issue of dosimetry and show how dosimetry tools enable to align data between different exposure systems or data from different laboratories and therefore allow comparisons to help further exploring the risk potential of e-cigarettes.

## Introduction

Vaping devices (*i.e.* electronic cigarettes or e-cigarettes) were introduced into the market a decade ago. They can be used as a possible alternative to tobacco smoking and some results showed that they can play an interesting role as a smoking cessation aid [1]. Besides, many studies seem to demonstrate that they are intrinsically less hazardous than conventional tobacco cigarettes and thus that health risks are expected to be less. However, concerns remain about their potential toxicity and adverse health effects, including long-term pulmonary or cardiovascular effects of the aerosol generated by e-cigarettes from the nicotine-containing fluids [2]. Indeed, instead of burning tobacco e-cigarette allows the heating of a so-called e-liquid, generating an aerosol that is inhaled by the vaper. The e-liquid is a mixture of variable composition including for instance propylene glycol, glycerol, water, various flavors and in most cases nicotine (although vapers can also use e-liquids without nicotine). While some of these compounds can exhibit an intrinsic toxicity, the heating process can also lead to the formation of new thermal decomposition compounds that may be also hazardous [2–4]. Approximately 250 chemical substances have already been detected in vaping aerosols, including substances initially present in the e-liquid formulation (*e.g.* nicotine, flavorings, propylene glycol), but also a large number of thermal degradation products (*e.g.* alkaloids, volatile organic compounds (VOCs), pyridine, carbonyl compounds such as acrolein or formaldehyde) and metals. In addition, thermal degradation substances of e-liquids such as tobacco-specific nitrosamines (TSNAs) and polycyclic aromatic hydrocarbons (PAHs) can also be found in some cases. The physical and chemical nature of vaping aerosols is quite different from burned tobacco smoke as no combustion process occurs during the vaporization of e-liquid. However, the particulate concentration in e-cigarette aerosol may be similar to that of tobacco cigarettes [3, 5, 6].

### Heterogeneity of studies

The toxicity of e-cigarette aerosol remains controversial, particularly because of the heterogeneity in the design of experimental toxicological studies regarding several parameters such as the choice of: (i) the e-cigarette products tested, (ii) the biological models used, and (iii) the different exposure conditions [3, 4, 7]. This heterogeneity makes it difficult to draw firm conclusions concerning the potential health adverse effect of vaping aerosol. Indeed, we cannot compare results obtained from such different experimental contexts, this makes meta-analyses impossible. Consequently, despite the wide amount of studies available in the literature, consistent comparisons cannot be made and robust and reliable conclusions cannot be drawn. However, although the diversity of study designs makes it difficult to draw quantitative conclusions, qualitative conclusions about health risks can be reached.

### Heterogeneity of e-cigarette products

The diversity of e-cigarette products (e-liquids formulations, and e-cigarette devices) available on the market and their constant high velocity evolution makes it challenging to test each product. Although sharing similar key components and operating procedures, e-cigarettes can be classified on four generations reflecting major differences in both their design and technology. First generation of e-cigarettes, also called “cig-a-like”, adequately mimic the appearance of conventional cigarettes [8]. These devices are mainly disposable, include little e-liquid and their inner components are not modifiable. Second generation e-cigarettes are reusable and introduce a refillable tank granting the user a better adjustment of nicotine exposure through the consumption of e-liquids containing variable nicotine concentrations [9]. The greatest flexibility is offered by the third generation e-cigarettes. These devices are based on a large refillable tank and an electronic card that directly controls the voltage and power. Because they are highly customizable, third generation e-cigarettes perfectly illustrate the heterogeneity of e-cigarette products and the complexity of comparative studies. Indeed, users can choose between various coil types (vertical, dual, triple, multiple, twisted, mesh, etc.), made of different alloys containing iron, chromium, aluminum or nickel and coils can be wicked with several materials including organic cotton balls, silica fibers wick, or Stainless Steel Mesh among others [10]. Moreover, a wide range of resistances is available on the market with values typically ranging from 0.1 to 3 ohms allowing the vaper to modify the density and temperature of the aerosol created. Finally, the most recent low-powered flash drive-shaped fourth generation e-cigarettes (*e.g.* Juul, NJOY, and Vuse) is mainly based on replaceable prefilled cartridge or POD containing nicotine salts that deliver much higher levels of nicotine than freebase nicotine [11]. Although these devices have not been designed to be modified by the user, closed cartridges can be easily dismounted and refilled with other e-liquids. Besides the heterogeneity of e-cigarettes devices, the vape market is also flooded with a tremendous diversity of e-liquids. In 2014, over 7500 different e-liquids were reported with approximately 250 new flavors released in the market per month [12]. These numbers rose to almost 20.000 e-liquids in the 2021 Dutch market [13] while a 2020 survey conducted in France revealed that nearly 27.000 e-liquids were available on the market [14]. Since screening toxicological studies are costly and time consuming, the huge heterogeneity of vape products and the significant amount of monthly released new products prevent an exhaustive assessment. Furthermore, comparison of studies using an identical device should be made carefully as several parameters can vary (*e.g.* power, resistance value, e-liquid formulation).

### Heterogeneity of biological models

While human clinical studies are available, the vast majority of studies aiming at investigating potential toxic effects of e-cigarettes use preclinical models such as cell culture and animal models [7]. Indeed, animal testing has traditionally been a primary method for evaluating product safety, however, it is expensive, time and personnel-consuming and most of all associated with ethical concerns [15–17]. In addition, criticisms about the translation of these results into clinical practice have been raised, for instance because of differences in the physiology and breathing behaviors between humans and rodents as well as the use of non-physiological exposure methods such as whole body exposure instead of mouth breathing [7, 18]. Probably the most important difference between experimental animals and humans is that rodents (since rats and mice are the species most often used) are obligatory nasal breathers while vaping is about oral inhalation. Rats and mice show the so-called “scrubbing effect” which means that a large part of inhaled substances will be deposited in the nasal passage and does not reach the lower respiratory tract. In parallel, with the development of new technologies, human relevant in vitro models for toxicity assessment have been proposed such as three dimensional (3D) advanced models or reconstructed human airway tissues [17, 18]. For these reasons, researchers massively turned their attention to in vitro alternative methods [16].

While too simplistic and not recapitulating neither the complexity of whole organisms nor the toxicokinetics [19] in vitro models offer many advantages. They permit different levels of study: organ, tissue, cell (one or several populations), they allow large screening of effects with a very small amount of test material and are very well adapted for the study of mechanisms, mainly for short term studies [17, 20, 21]. As they are performed under controlled testing conditions they allow reduction of variability between experiments [20].

The effects of e-cigarettes have been assessed using a wide range of target cells, especially from the respiratory tract [7]. A non-exhaustive list of the diversity of cellular models is shown in Table 1. It also illustrates the variety of biological endpoints assessed, adding a supplementary layer of heterogeneity and complexity in cross-analyses of results.

**Table 1:** Diversity of the cellular models used in e-cigarette toxicological studies (not exhaustive)

Cell line	Anatomical location	Cell type	Biological endpoints	References
MSK-Leuk1	Oral	Cancerous from dysplastic leukoplakia near the tongue	Cell viability Oxidative stress Genotoxicity	[22]
EpiOral™	Oral	Commercially available primary oral model from MatTek	Histology Inflammatory mediators Transcriptomic	[23]
Human primary nasal epithelial cells	Nasal	Primary cells from never-smoker patient	Bacterial adhesion	[24]
Human primary airway epithelial nasal cells	Nasal	Commercially available primary cells from Epithelix	Cell viability Inflammatory cytokines Mucins expression	[25]
Oropharyngeal mucosa	Pharynx	Primary cells from healthy patients	Cell viability Genotoxicity	[26]
Calu-3	Bronchial	Cancerous from lung adenocarcinoma	Inflammatory cytokines Bacterial adhesion	[27]
NCI-H292	Bronchial	Cancerous from pulmonary mucoepidermoid carcinoma	Oxidative stress Inflammatory cytokines Tight junction integrity	[28]
16HBE14o-	Bronchial	SV40-immortalized normal bronchial epithelial cells	Cell viability Tight junction integrity	[29]
BEAS-2B	Bronchial	Normal bronchial epithelial cells	Oxidative stress Inflammatory cytokines Transcriptomic analysis	[30]
CL-1548	Bronchial	hTERT-immortalized primary bronchial epithelial cells	Cell viability Oxidative stress	[31]
NHBE	Bronchial	Primary bronchial epithelial cells from healthy donors	Cell viability Oxidative stress Apoptosis	[32]
MucilAir™	Bronchial	Commercially available primary bronchial model from Epithelix	Tight junction integrity Ciliary beat frequency Permeability	[33]
EpiAirway™	Bronchial	Commercially available primary bronchial model from MatTek	Cell viability Tight junction integrity	[34]
SmallAir™	Bronchial—small airway	Commercially available primary small airway model from Epithelix	Histology Inflammatory mediators Transcriptomic analysis Ciliary beat frequency	[23]
A549	Alveolar	Cancerous from lung adenocarcinoma	Cell viability Apoptosis Genotoxicity	[35]
Alveolar Macrophages	Immune system	Primary cells from healthy patients	Phagocytosis Apoptosis/Necrosis Inflammatory cytokines Oxidative stress	[36]
U937	Immune system	Cancerous monocytes from pleural effusion	Cell viability Oxidative stress Inflammatory cytokines	[37]

It could be surprising to find nasal models as vaping is about oral inhalation. However, many vapers exhibit a practice in which e-cigarette aerosols are frequently exhaled through the nose (simulating a habit that is also very common among tobacco smokers). Furthermore, regardless of how the vaper exhales, a fraction of the exhaled aerosol comes into contact with the nasal cavities. Thus, nasal cell models may be relevant to assess the health risk of vaping.

A single cell type can be used or alternatively co-cultures of different cell types, which include cell interactions and better reproduce a physiological micro-environment [18]. A widely used model for inhalation toxicity evaluation is 3-dimensional (3D) in vitro models ranging from spheroids and organoids to the reproduction of an organotypic tissue. Some models are commercially available such as EpiAirway™, constructed from primary human tracheal-bronchial epithelial cells that form a fully differentiated, pseudostratified epithelium containing mucus-producing goblet cells, ciliated cells and basal cells [16]. These models are then exposed at the air–liquid interface (ALI), providing a useful platform for the toxicity assessment of e-cigarette aerosols [16]. Furthermore, they seem to have the potential to be more predictive of effects in humans since they are derived from biopsied human cells and contain many of the relevant differentiated cell types not found in monolayer cultures. As an example, EpiAirway™ tissues have been shown to be quite predictive of in vivo respiratory response to chemicals, although the comparisons were made only with a classification based on in vivo rat studies and not humans [38]. Even if this comparison is promising, this is a definite limitation that does not allow for a robust evidence of excellent in vivo prediction of EpiAirway™ tissues in humans which is still quite far from prediction in the sense of a quantitative estimate of health risks from long term daily e-cigarette use by humans.

It is well known that increasing the complexity of in vitro models improve both their physiological relevance and robustness of the generated data. However, these benefits are balanced by a high cost and an increased variability. Thus, the choice of a cell model should be carefully adapted to the design of the study and the biological endpoints [39]. Overall, well-characterized immortalized cell lines are a great tool for low-cost large screening studies investigating a wide set of products focused on basic biological endpoints as cell viability, oxidative stress or inflammatory cytokines released. On the other hand, complex 3D models are useful to assess tissue-specific markers (mucus production, ciliary beat frequency, surfactant composition, etc.) and in-depth molecular mechanisms as well as to conduct chronic exposure studies.

The heterogeneity of biological models observed in e-cigarette toxicological studies makes it difficult to compare data across studies as a result of two main issues. The first issue is the diversity in anatomical locations of cell models used. Because of the phenotypic variability among cell populations composing the respiratory tract, biological responses following e-cigarette aerosol exposure will differ between models from different anatomical locations. A transcriptomic analysis between primary oral and bronchial models following e-cigarette aerosol exposure showed that bronchial epithelium was more sensitive than oral tissue but recovered quicker suggesting a tissue-specific response through distinct molecular pathways [23]. The second issue is related to the susceptibility towards chemical substances which can vary depending on the cell type. CL-1548 primary immortalized bronchial cells were less sensitive to e-cigarette aerosol compared to non-immortalized bronchial cells NHBE [31]. Similarly, H292 cancerous bronchial cells were less susceptible than normal BEAS-2B bronchial cells in terms of toxicity and gene expression [28]. This is a limitation to keep in mind when working with immortalized cell lines. Indeed, while primary cultures may better mimic a physiological behavior, they are associated with technical challenges (lack of tissue availability, specific handling required and donor-specific variations) [19, 40]. Usually, cells lines are preferred because of their homogeneity and stability resulting in reproducible results. However, they are either cancer cells or cells artificially immortalized and although their high proliferative rate makes them easily cultivable, available in large quantities, and inexpensive, they exhibit altered pathways compared to normal cells. Consequently, they should not answer exactly as healthy cells would do, making them a poorly reliable representation of what really occurs in vivo. In addition, if they are used for long periods of time, a cell de-differentiation and thus a change in phenotype can occur [17, 19, 40].

### Heterogeneity of exposure conditions

The cytotoxicity of vaping commercial products is usually assessed as e-liquids and/or as aerosols [6, 7]. Exposure of cells (submerged culture) to unheated e-liquid or to one of its specific ingredients is a very inexpensive, simple and high-throughput method for rapid screening. However, we should keep in mind that most of the components of the e-liquid are changed through aerosolization and that the complex gas mixture from heating process is mainly responsible for the cytotoxic effects [18]. But it is not just the change of chemicals as a result of heating that makes the composition of the aerosol different from that of the e-liquid. It is also simply a matter of volatility that makes that some components evaporate more quickly than others. Submerged cells can alternatively be exposed to an aqueous extract consisting of an aerosol that has been bubbled through media or buffer. This is a relatively simple and inexpensive exposure that captures both the water-soluble particulate and gas phase components [18]. Despite a good representation of e-cigarette aerosol composition, this method only captures cell medium soluble compounds. The latter can further cross-react with proteins in cell medium which may reduce their bioavailability and underestimate their toxicity. Lastly, aqueous extracts are not suitable to ALI cell models since the application of these extracts is based on cell submersion, a non-physiological exposure method that annihilates the benefits of ALI. To avoid bias related to e-cigarette aerosol capture, more complex exposure systems involve the direct exposure of cells to a whole aerosol at the air–liquid interface. Although more challenging and costly, ALI better recapitulates physiological conditions of the pulmonary barrier and all phases (gaseous, semi-volatile and particulate) and components of the test aerosol are included in the analysis [18].

The physiological limitations of in vitro assays based on e-cigarette extracts or condensates led to the development of new exposure systems (or the adaptation of existing ones) for exposure of cell models at the air–liquid interface. Aerosol exposure systems have been historically used for the characterization of conventional cigarette smoke and extensively reviewed elsewhere [41, 42]. These systems were later adapted and characterized for e-cigarette aerosols. Exposure systems are composed of two distinct modules, a smoking machine generating e-cigarette aerosols connected to an exposure chamber that contains and maintains the cells at ALI [42]. The most described references are the Vitrocell VC 10 Smoking Robot and the Borgwaldt RM20S Smoking machine. Although designed for a same goal, VC10 and RM20S have been shown to deliver significantly different deposited masses and nicotine concentrations for an identical puffing regimen [43]. This was attributed to differences in aerosol sampling/dilution system, transit length and exposure chambers. Indeed, the RM20S was connected to a passive exposure chamber (gravitational settling of aerosol) while VC10 was paired to a Vitrocell 6/4 exposure module in which the aerosol is actively guided toward the cells by negative pressure. A recent study compared the e-cigarette-designed Linear E-cigarette puffing Machine (LM4E) with a modified VC10 to investigate the toxicity of undiluted e-cigarette aerosol [44]. Interestingly, authors showed that, despite significant differences in nicotine concentration, cytotoxic profile was consistent between the two systems. This important result showed that the use of undiluted aerosols could facilitate comparisons of e-cigarette toxicological data between different exposure systems by mitigating the variability induced by the dilution step. Another low-cost exposure chamber suitable for undiluted aerosol is the Vitrocell CLOUD in which aerosol is directly introduced from a nebulizer before uniformly sedimenting by gravitational settling [45]. However, this easy handling system remains to be characterized in a complete setup dedicated for e-cigarettes aerosol exposure using an appropriate puffing regimen. Besides Vitrocell and Borgwaldt, several manufacturers have developed others smoking machine/exposure chamber (Burghardt, CULTEX, Philip Morris, British American Tobacco…) with various engineering processes and technologies [46]. Taken together, the heterogeneity of both exposure protocols and exposure systems strengthens the difficulty of comparing data obtained from e-cigarette toxicological studies and to draw robust conclusions concerning e-cigarette health effects. Interestingly, a case study showed that biological response to cigarette smoke can be compared across five different systems if datasets are normalized as a function of dose (µg/cm^2^) and nicotine concentration (mg) [47]. This important work suggests that adequate in vitro dosimetry techniques are required and can effectively unit data between various setups and exposure protocols.

### Dosimetry, a major issue

#### Different ways to express doses and different dose ranges used

When considering the studies dealing with the toxicity of e-cigarettes assessment, in addition to the heterogeneity of e-cigarette products, biological models, or exposure conditions observed, there is also a huge disparity in the doses used as well as in the way they are expressed as shown in Table 2.

**Table 2:** Examples of different metrics and dose ranges used in studies for the assessment of the toxicity of e-cigarette (not exhaustive)

Dose metric	Dose range	Reference
Number of puffs (puff volume)	10–60 puffs (55 mL) 20 puffs (166 mL) 0–35 puffs (70 mL) 0–400 puffs (55 mL) 0–900 puffs (55 mL)	[48] [49] [50, 51] [16] [52]
Puffs/mL*	0–0.5 puffs/mL 0.075–0.75 puffs/mL 0.02–6 puffs/mL 0–280 puffs/L	[53] [54] [5, 55] [56]
Puffs/m^2^	85 and 257 puffs/m^2^	[57]
Concentration of a compound** (µM; mM; mg/mL)	10–1000 µM flavor 1–5 mM flavor 0–600 mg/mL heptyl butyrate, methyl stearate, butyl methacrylate and heptanal 0–10 mg/mL for diverse compounds including flavors	[37, 58] [59] [34] [60]
Ratio (%), corresponding to the dilution of e-liquid	0.001–1% 0–0.3% 0–1% 0–3% 0–4% 0–10% 0–30%	[61, 62] [63] [60] [50] [64] [55, 65] [51]
Ratio (%), corresponding to the dilution of a cigarette or aerosol extract	0.625–20% 3.125–100% 12.5–100% 40–100%	[66] [67, 68] [69] [70]

*Puffs/mL corresponds to the number of puffs reported to the volume of solvent in which the aerosol was captured. **Concentration of a compound diluted in the culture medium and used in in vitro assays

#### Do we have to use physiological doses?

The wide variety of dose ranges used leads to the question of the most relevant doses to use when assessing the toxicity of e-cigarettes. Do we have to use physiological doses to more closely mimic what could happen in vivo?

As mentioned before, in vitro models are very well adapted for mechanistic studies. Therefore, in addition to exposure doses that tend to mimic the real-life conditions of e-cigarette use, unrealistically high doses can also be used without being fundamentally objectionable if the ultimate goal is to demonstrate dose-dependent effects that are very helpful in terms of elucidating mechanistic aspects of the toxicological process, even though such doses would neither be applicable nor would make sense in in vivo studies, and thus require special attention when interpreting the results. Indeed, the use of extreme conditions increases the likelihood of eliciting a response, potentially allowing for small effects to be observed that would otherwise be missed or represented as no result and also allowing comparisons to combustible cigarettes via EC_50_ values. However, when using this approach, experimental exposure condition should be contextualized to real-life usage and care should be taken to not over-extrapolate conclusions [71].

**Fig. 1:**
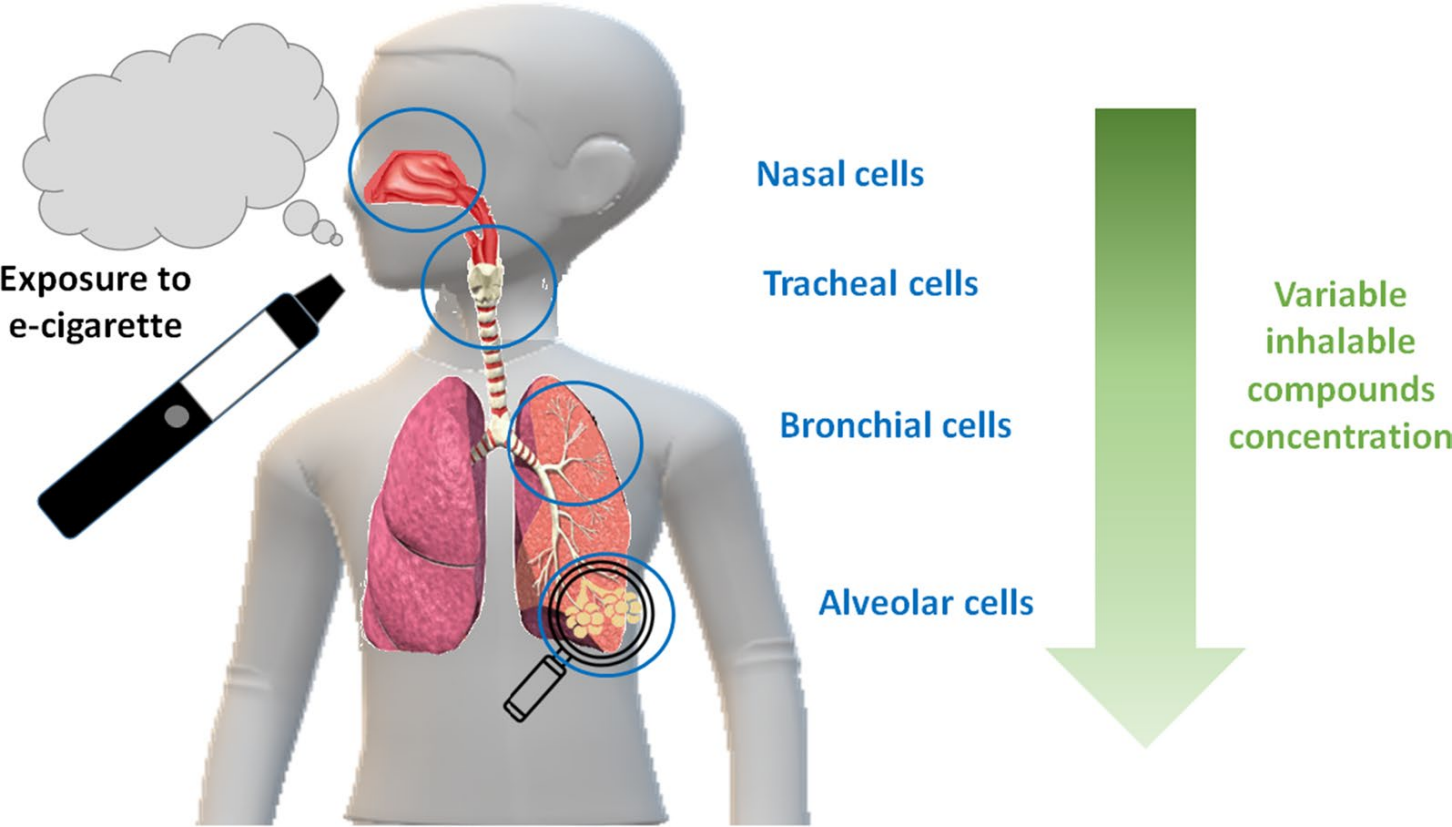
Variable dose of inhalable materials depending on the anatomical location within the respiratory tract

However, when one wants to compare the in vitro effects of e-cigarettes on different biological targets, the dose should be adapted as illustrated by Fig. 1. Let’s take the example of a study of the impact of e-cigarette at different levels of the respiratory tract, *i.e.* from the upper respiratory airways (nasal cells) to the deepest lung (alveolar cells). Using the same dose range for the different cell types would not be relevant as they are differentially exposed to the inhaled compounds. In other words, the dose actually reaching the target cells can vary considerably depending on the target area considered. Indeed, different deposition mechanisms, such as diffusion, sedimentation, inertial impaction, interception and electrostatic forces, can affect the deposition of inhaled droplets in the respiratory system [18, 72]. In this regard, a Multiple Path Particle Dosimetry (MPPD) model could be used to predict deposition dose at the different locations of the human (and rat) respiratory tract. This may be helpful to choose the range of exposure doses in in vitro assays, but also for the interpretation of the in vitro test results to the in vivo situation.

Thus if we want to be consistent and relevant to physiological deposition we should adapt the concentration range to the cell model. In the example, nasal cells should be exposed to higher concentrations while alveolar cells should be exposed to lower concentrations as due to the anatomical location, less compounds will reach such structures.

**Fig. 2:**
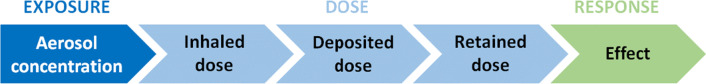
The dose reaching and effectively retained in a target is different from the inhaled dose

As the mechanism of toxicity and the severity of toxicity outcome highly depend on the dose, it is crucial to characterize the actual dose reaching the target (Fig. 2). The knowledge of the delivered dose and its time course using reliable dosimetry techniques is critical to interpreting and potentially extrapolating results from in vitro assays, but also to gain in homogeneity and be able to compare different toxicological studies [18].

### Dosimetry approaches for standardization

Especially in the case of comparative studies such as the effects of e-cigarette versus those of conventional cigarettes, to characterize dosimetry and normalize results we can use chemical markers common to cigarette and e-cigarette aerosol. Among them, nicotine is extensively used since it is an ingredient common to cigarettes and e-cigarettes with well-documented addiction promoting activity and toxicity [18, 27, 43].

Dosimetry, *i.e.* the quantification of what the cell cultures are exposed to at the interface, is thus crucial to the accurate biological assessment of the effects of the exposure to different aerosols [43, 71]. Indeed, the deposited aerosol from a conventional cigarette and an e-cigarette are compositionally different. The quantification of the dose is also crucial to accurately compare e-cigarette devices since significant variations in the deposited mass were observed between two devices using an identical puffing profile [71]. Dosimetry tools enable easier extrapolation and comparison of pre-clinical data and consumer use studies, to help further explore the reduced risk potential of next generation nicotine products [43, 73]. To that purpose, a widely used method is the measurement of the deposited mass with the quartz crystal microbalance (QCM), which is placed inside a cell exposure chamber at the cell–aerosol interface. QCM is very sensitive as it can detect changes in mass within the nanogram range, and allow measurement of deposited mass per surface area (µg/cm^2^) in real time. The characterization of the deposited mass allows direct comparison of mass-based dose versus biological responses to aerosols, which is important for toxicity and safety assessment studies. It also serves as a quality control confirming within an exposure that the culture in the exposure chamber is indeed receiving the aerosol dilution that is being reported [43, 71, 73]. Using this approach Adamson et al. were able to determine if a differential cytotoxic response could be elicited from exposure of an in vitro human bronchial epithelial model to aerosols generated from an e-cigarette and a conventional cigarette [43]. Although QCM can be considered as a reliable dosimetry tool, care should be taken concerning the reported mass. Indeed, QCM was shown to dramatically underestimate the deposited mass of a glycerol-based model aerosol compared to the fluorometric quantification of a tracer [74]. These differences could be explained by the viscosity of the aerosol and the deposited liquid layer that may impair the resonant frequency of the quartz to ultimately bias the QCM readout. Thus, further studies should ensure the reliability of QCM measurement including other quantification methods. Among them, nicotine is a reliable in situ cross-product dosimetric marker [52] while gravimetric method based on Cambridge filters weighing could be alternatively used when working with nicotine-free e-liquids [75]. Finally, it is worth mentioning that the combination of several dosimetry methods within a study could dramatically increase the relevance of toxicological data in addition to readily enable comparison of the results with other works.

## Conclusion

The lack of standardization among the studies aiming to assess in vitro e-cigarette toxicity makes it challenging to conclude on their safety [4]. This heterogeneity regards the e-cigarette products, the biological models, the exposure systems, and also the doses used. Because of this diversity, although e-cigarettes seem to appear less harmful than tobacco cigarettes, their long term adverse health effects on humans cannot be easily predicted from the currently available data [2, 7]. Therefore the development of a standardized approach to evaluate the e-cigarette toxicity is doubtless an urgent need [6]. In this approach, dosimetry should be carefully considered to align experimental data generated from completely different exposure systems or conditions, or data from different laboratories. In addition, although some limitations remain, dosimetry could provide an helpful link between in vitro*, *in vivo and human studies and thus greatly facilitate the comparison of data across different categories of tobacco and nicotine products [18, 71].

## Data Availability

Not applicable.

## References

[1] Hajek P, Phillips-Waller A, Przulj D, Pesola F, Myers Smith K, Bisal N (2019). A randomized trial of e-cigarettes versus nicotine-replacement therapy. N Engl J Med.

[2] Marques P, Piqueras L, Sanz M-J (2021). An updated overview of e-cigarette impact on human health. Respir Res.

[3] Dinakar C, O’Connor GT (2016). The health effects of electronic cigarettes. N Engl J Med.

[4] Cao Y, Wu D, Ma Y, Ma X, Wang S, Li F (2021). Toxicity of electronic cigarettes: a general review of the origins, health hazards, and toxicity mechanisms. Sci Total Environ.

[5] Behar RZ, Wang Y, Talbot P (2018). Comparing the cytotoxicity of electronic cigarette fluids, aerosols and solvents. Tob Control.

[6] Wang G, Liu W, Song W (2019). Toxicity assessment of electronic cigarettes. Inhal Toxicol.

[7] Hiemstra PS, Bals R (2016). Basic science of electronic cigarettes: assessment in cell culture and in vivo models. Respir Res.

[8] Trtchounian A, Talbot P (2011). Electronic nicotine delivery systems: is there a need for regulation?. Tob Control.

[9] Hess CA, Olmedo P, Navas-Acien A, Goessler W, Cohen JE, Rule AM (2017). E-cigarettes as a source of toxic and potentially carcinogenic metals. Environ Res.

[10] Snoderly HT, Nurkiewicz TR, Bowdridge EC, Bennewitz MF (2021). E-cigarette use: device market, study design, and emerging evidence of biological consequences. Int J Mol Sci.

[11] Jackler RK, Ramamurthi D (2019). Nicotine arms race: JUUL and the high-nicotine product market. Tob Control.

[12] Zhu S-H, Sun JY, Bonnevie E, Cummins SE, Gamst A, Yin L (2014). Four hundred and sixty brands of e-cigarettes and counting: implications for product regulation. Tob Control.

[13] Havermans A, Krüsemann EJZ, Pennings J, de Graaf K, Boesveldt S, Talhout R (2021). Nearly 20 000 e-liquids and 250 unique flavour descriptions: an overview of the Dutch market based on information from manufacturers. Tob Control.

[14] Anses. RAPPORT de l’Anses relatif à la déclaration des produits du tabac et des produits connexes en France—Produits du vapotage—Bilan 2016–2020 [Internet]. 2020. Available from: https://www.anses.fr/fr/content/rapport-de-lanses-relatif-%C3%A0-la-d%C3%A9claration-des-produits-du-tabac-et-des-produits-connexes-en.

[15] Kumar V, Sharma N, Maitra SS (2017). In vitro and in vivo toxicity assessment of nanoparticles. Int Nano Lett.

[16] Czekala L, Simms L, Stevenson M, Tschierske N, Maione AG, Walele T (2019). Toxicological comparison of cigarette smoke and e-cigarette aerosol using a 3D in vitro human respiratory model. Regul Toxicol Pharmacol.

[17] Forest V (2022). Experimental and computational nanotoxicology—complementary approaches for nanomaterial hazard assessment. Nanomaterials.

[18] Behrsing H, Hill E, Raabe H, Tice R, Fitzpatrick S, Devlin R (2017). In vitro exposure systems and dosimetry assessment tools for inhaled tobacco products: workshop proceedings, conclusions and paths forward for in vitro model use. Altern Lab Anim.

[19] Joris F, Manshian BB, Peynshaert K, De Smedt SC, Braeckmans K, Soenen SJ (2013). Assessing nanoparticle toxicity in cell-based assays: influence of cell culture parameters and optimized models for bridging the in vitro-in vivo gap. Chem Soc Rev.

[20] Takhar P, Mahant S. In vitro methods for nanotoxicity assessment: advantages and applications. Arch Appl Sci Res. 2011;3.

[21] Fröhlich E, Salar-Behzadi S (2014). Toxicological assessment of inhaled nanoparticles: role of in vivo, ex vivo, in vitro, and in silico studies. Int J Mol Sci.

[22] Tellez CS, Juri DE, Phillips LM, Do K, Yingling CM, Thomas CL (2021). Cytotoxicity and genotoxicity of e-cigarette generated aerosols containing diverse flavoring products and nicotine in oral epithelial cell lines. Toxicol Sci.

[23] Iskandar AR, Zanetti F, Kondylis A, Martin F, Leroy P, Majeed S (2019). A lower impact of an acute exposure to electronic cigarette aerosols than to cigarette smoke in human organotypic buccal and small airway cultures was demonstrated using systems toxicology assessment. Intern Emerg Med.

[24] Miyashita L, Suri R, Dearing E, Mudway I, Dove RE, Neill DR (2018). E-cigarette vapour enhances pneumococcal adherence to airway epithelial cells. Eur Respir J.

[25] Kwak S, Choi YS, Na HG, Bae CH, Song S-Y, Kim Y-D (2021). Glyoxal and methylglyoxal as e-cigarette vapor ingredients-induced pro-inflammatory cytokine and mucins expression in human nasal epithelial cells. Am J Rhinol Allergy.

[26] Welz C, Canis M, Schwenk-Zieger S, Becker S, Stucke V, Ihler F (2016). Cytotoxic and genotoxic effects of electronic cigarette liquids on human mucosal tissue cultures of the oropharynx. J Environ Pathol Toxicol Oncol.

[27] Herr C, Tsitouras K, Niederstraßer J, Backes C, Beisswenger C, Dong L (2020). Cigarette smoke and electronic cigarettes differentially activate bronchial epithelial cells. Respir Res.

[28] Pinkston R, Zaman H, Hossain E, Penn AL, Noël A (2020). Cell-specific toxicity of short-term JUUL aerosol exposure to human bronchial epithelial cells and murine macrophages exposed at the air-liquid interface. Respir Res.

[29] Sherwood CL, Boitano S (2016). Airway epithelial cell exposure to distinct e-cigarette liquid flavorings reveals toxicity thresholds and activation of CFTR by the chocolate flavoring 2,5-dimethypyrazine. Respir Res.

[30] Anthérieu S, Garat A, Beauval N, Soyez M, Allorge D, Garçon G (2017). Comparison of cellular and transcriptomic effects between electronic cigarette vapor and cigarette smoke in human bronchial epithelial cells. Toxicol In Vitro.

[31] Scheffler S, Dieken H, Krischenowski O, Aufderheide M (2015). Cytotoxic evaluation of e-liquid aerosol using different lung-derived cell models. Int J Environ Res Public Health.

[32] Czekala L, Simms L, Stevenson M, Trelles-Sticken E, Walker P, Walele T (2019). High content screening in NHBE cells shows significantly reduced biological activity of flavoured e-liquids, when compared to cigarette smoke condensate. Toxicol In Vitro.

[33] Ghosh B, Reyes-Caballero H, Akgün-Ölmez SG, Nishida K, Chandrala L, Smirnova L (2020). Effect of sub-chronic exposure to cigarette smoke, electronic cigarette and waterpipe on human lung epithelial barrier function. BMC Pulm Med.

[34] Neilson L, Mankus C, Thorne D, Jackson G, DeBay J, Meredith C (2015). Development of an in vitro cytotoxicity model for aerosol exposure using 3D reconstructed human airway tissue; application for assessment of e-cigarette aerosol. Toxicol In Vitro.

[35] Khalil C, Chahine JB, Haykal T, Al Hageh C, Rizk S, Khnayzer RS (2021). E-cigarette aerosol induced cytotoxicity, DNA damages and late apoptosis in dynamically exposed A549 cells. Chemosphere.

[36] Scott A, Lugg ST, Aldridge K, Lewis KE, Bowden A, Mahida RY (2018). Pro-inflammatory effects of e-cigarette vapour condensate on human alveolar macrophages. Thorax.

[37] Muthumalage T, Prinz M, Ansah KO, Gerloff J, Sundar IK, Rahman I (2017). Inflammatory and oxidative responses induced by exposure to commonly used e-cigarette flavoring chemicals and flavored e-liquids without nicotine. Front Physiol.

[38] Jackson GR, Maione AG, Klausner M, Hayden PJ (2018). Prevalidation of an acute inhalation toxicity test using the EpiAirway In vitro human airway model. Appl In Vitro Toxicol.

[39] Lacroix G, Koch W, Ritter D, Gutleb AC, Larsen ST, Loret T (2018). Air-liquid interface in vitro models for respiratory toxicology research: consensus workshop and recommendations. Appl In Vitro Toxicol.

[40] Drasler B, Sayre P, Steinhäuser KG, Petri-Fink A, Rothen-Rutishauser B (2017). In vitro approaches to assess the hazard of nanomaterials. NanoImpact.

[41] Thorne D, Adamson J (2013). A review of in vitro cigarette smoke exposure systems. Exp Toxicol Pathol.

[42] Li X (2016). In vitro toxicity testing of cigarette smoke based on the air-liquid interface exposure: a review. Toxicol In Vitro.

[43] Adamson J, Thorne D, Zainuddin B, Baxter A, McAughey J, Gaça M (2016). Application of dosimetry tools for the assessment of e-cigarette aerosol and cigarette smoke generated on two different in vitro exposure systems. Chem Cent J.

[44] Bishop E, Terry A, East N, Breheny D, Gaça M, Thorne D (2022). A 3D in vitro comparison of two undiluted e-cigarette aerosol generating systems. Toxicol Lett.

[45] Nair V, Tran M, Behar RZ, Zhai S, Cui X, Phandthong R (2020). Menthol in electronic cigarettes: a contributor to respiratory disease?. Toxicol Appl Pharmacol.

[46] Thorne D, Adamson J, Sticken ET, Wieczorek R, Behrsing H, Steiner S (2021). An interlaboratory in vitro aerosol exposure system reference study. Toxicol Res Appl..

[47] Thorne D, Bishop E, Haswell L, Gaça M (2018). A case study for the comparison of in vitro data across multiple aerosol exposure studies with extrapolation to human dose. Appl In Vitro Toxicol.

[48] Caruso M, Distefano A, Emma R, Zuccarello P, Copat C, Ferrante M, et al. In vitro cytoxicity profile of e-cigarette liquid samples on primary human bronchial epithelial cells. Drug Test Anal. 2022; 10.1002/dta.3275 35434934

[49] Escobar Y-NH, Nipp G, Cui T, Petters SS, Surratt JD, Jaspers I (2020). In vitro toxicity and chemical characterization of aerosol derived from electronic cigarette humectants using a newly developed exposure system. Chem Res Toxicol.

[50] Rowell TR, Reeber SL, Lee SL, Harris RA, Nethery RC, Herring AH (2017). Flavored e-cigarette liquids reduce proliferation and viability in the CALU3 airway epithelial cell line. Am J Physiol Lung Cell Mol Physiol.

[51] Sassano MF, Davis ES, Keating JE, Zorn BT, Kochar TK, Wolfgang MC (2018). Evaluation of e-liquid toxicity using an open-source high-throughput screening assay. PLoS Biol.

[52] Thorne D, Hollings M, Seymour A, Adamson J, Dalrymple A, Ballantyne M (2018). Extreme testing of undiluted e-cigarette aerosol in vitro using an Ames air-agar-interface technique. Mutat Res Genet Toxicol Environ Mutagen.

[53] Taylor M, Carr T, Oke O, Jaunky T, Breheny D, Lowe F (2016). E-cigarette aerosols induce lower oxidative stress in vitro when compared to tobacco smoke. Toxicol Mech Methods.

[54] Abouassali O, Chang M, Chidipi B, Martinez JL, Reiser M, Kanithi M (2021). In vitro and in vivo cardiac toxicity of flavored electronic nicotine delivery systems. Am J Physiol Heart Circ Physiol.

[55] Omaiye EE, McWhirter KJ, Luo W, Pankow JF, Talbot P (2019). High-nicotine electronic cigarette products: toxicity of JUUL fluids and aerosols correlates strongly with nicotine and some flavor chemical concentrations. Chem Res Toxicol.

[56] Wang H, Chen H, Huang L, Li X, Wang L, Li S (2021). In vitro toxicological evaluation of a tobacco heating product THP COO and 3R4F research reference cigarette on human lung cancer cells. Toxicol In Vitro.

[57] Marrocco A, Singh D, Christiani DC, Demokritou P (2022). E-Cigarette (E-Cig) liquid composition and operational voltage define the in vitro toxicity of Δ8Tetrahydrocannabinol/Vitamin E acetate (Δ8THC/VEA) E-Cig aerosols. Toxicol Sci.

[58] Morris AM, Leonard SS, Fowles JR, Boots TE, Mnatsakanova A, Attfield KR (2021). Effects of e-cigarette flavoring chemicals on human macrophages and bronchial epithelial cells. Int J Environ Res Public Health.

[59] Jabba SV, Diaz AN, Erythropel HC, Zimmerman JB, Jordt S-E (2020). Chemical adducts of reactive flavor aldehydes formed in e-cigarette liquids are cytotoxic and inhibit mitochondrial function in respiratory epithelial cells. Nicotine Tob Res.

[60] Hua M, Omaiye EE, Luo W, McWhirter KJ, Pankow JF, Talbot P (2019). Identification of cytotoxic flavor chemicals in top-selling electronic cigarette refill fluids. Sci Rep.

[61] Bahl V, Lin S, Xu N, Davis B, Wang Y, Talbot P (2012). Comparison of electronic cigarette refill fluid cytotoxicity using embryonic and adult models. Reprod Toxicol.

[62] Behar RZ, Davis B, Wang Y, Bahl V, Lin S, Talbot P (2014). Identification of toxicants in cinnamon-flavored electronic cigarette refill fluids. Toxicol In Vitro.

[63] Wu Q, Jiang D, Minor M, Chu HW (2014). Electronic cigarette liquid increases inflammation and virus infection in primary human airway epithelial cells. PLoS ONE.

[64] Iskandar AR, Gonzalez-Suarez I, Majeed S, Marescotti D, Sewer A, Xiang Y (2016). A framework for in vitro systems toxicology assessment of e-liquids. Toxicol Mech Methods.

[65] Go YY, Mun JY, Chae S-W, Chang J, Song J-J (2020). Comparison between in vitro toxicities of tobacco- and menthol-flavored electronic cigarette liquids on human middle ear epithelial cells. Sci Rep.

[66] Rankin GD, Wingfors H, Uski O, Hedman L, Ekstrand-Hammarström B, Bosson J (2019). The toxic potential of a fourth-generation E-cigarette on human lung cell lines and tissue explants. J Appl Toxicol.

[67] Romagna G, Allifranchini E, Bocchietto E, Todeschi S, Esposito M, Farsalinos KE (2013). Cytotoxicity evaluation of electronic cigarette vapor extract on cultured mammalian fibroblasts (ClearStream-LIFE): comparison with tobacco cigarette smoke extract. Inhal Toxicol.

[68] Farsalinos KE, Romagna G, Allifranchini E, Ripamonti E, Bocchietto E, Todeschi S (2013). Comparison of the cytotoxic potential of cigarette smoke and electronic cigarette vapour extract on cultured myocardial cells. Int J Environ Res Public Health.

[69] Leslie LJ, Vasanthi Bathrinarayanan P, Jackson P, Mabiala Ma Muanda JA, Pallett R, Stillman CJP (2017). A comparative study of electronic cigarette vapor extracts on airway-related cell lines in vitro. Inhal Toxicol.

[70] Taylor M, Jaunky T, Hewitt K, Breheny D, Lowe F, Fearon IM (2017). A comparative assessment of e-cigarette aerosols and cigarette smoke on in vitro endothelial cell migration. Toxicol Lett.

[71] Azzopardi D, Patel K, Jaunky T, Santopietro S, Camacho OM, McAughey J (2016). Electronic cigarette aerosol induces significantly less cytotoxicity than tobacco smoke. Toxicol Mech Methods.

[72] Forest V, Pourchez J (2022). Nano-delivery to the lung—by inhalation or other routes and why nano when micro is largely sufficient?. Adv Drug Deliv Rev.

[73] Thorne D, Larard S, Baxter A, Meredith C, Gaҫa M (2017). The comparative in vitro assessment of e-cigarette and cigarette smoke aerosols using the γH2AX assay and applied dose measurements. Toxicol Lett.

[74] Steiner S, Majeed S, Kratzer G, Vuillaume G, Hoeng J, Frentzel S (2017). Characterization of the Vitrocell® 24/48 aerosol exposure system for its use in exposures to liquid aerosols. Toxicol In Vitro.

[75] Zhang J, Doshi U, Wolz RL, Kosachevsky P, Oldham MJ, Gillman IG (2022). Fit-for-purpose characterization of air-liquid-interface (ALI) in vitro exposure systems for e-vapor aerosol. Toxicol In Vitro.

